# Convergent abnormalities in striatal gene networks in human cocaine use disorder and mouse cocaine administration models

**DOI:** 10.1126/sciadv.add8946

**Published:** 2023-02-10

**Authors:** Philipp Mews, Ashley M. Cunningham, Joseph Scarpa, Aarthi Ramakrishnan, Emily M. Hicks, Sarah Bolnick, Susanna Garamszegi, Li Shen, Deborah C. Mash, Eric J. Nestler

**Affiliations:** ^1^ Nash Family Department of Neuroscience and Friedman Brain Institute, Icahn School of Medicine at Mount Sinai, New York, NY, USA.; ^2^ Department of Anesthesiology, Weill Cornell Medical College, New York, NY, USA.; ^3^ Pamela Sklar Division of Psychiatric Genomics, Icahn School of Medicine at Mount Sinai, New York, NY, USA.; ^4^ Department of Neurology, Miller School of Medicine, University of Miami, Miami, FL, USA.

## Abstract

Cocaine use disorder (CUD) is an intractable syndrome, and rising overdose death rates represent a substantial public health crisis that exacts tremendous personal and financial costs on patients and society. Sharp increases in cocaine use drive the urgent need for better mechanistic insight into this chronic relapsing brain disorder that currently lacks effective treatment options. To investigate the transcriptomic changes involved, we conducted RNA sequencing on two striatal brain regions that are heavily implicated in CUD, the nucleus accumbens and caudate nucleus, from men suffering from CUD and matched controls. Weighted gene coexpression analyses identified CUD-specific gene networks enriched in ionotropic receptors and linked to lowered neuroinflammation, contrasting the proinflammatory responses found in opioid use disorder. Integration of comprehensive transcriptomic datasets from mouse cocaine self-administration models revealed evolutionarily conserved gene networks in CUD that implicate especially D1 medium spiny neurons as drivers of cocaine-induced plasticity.

## Teaser

Cocaine use disorder alters evolutionarily conserved striatal gene networks of neuroinflammation and ionotropic receptors.

## INTRODUCTION

Cocaine use disorder (CUD) is a major public health problem that is associated with substantial morbidity and mortality. Within the United States, the rate of cocaine overdose deaths has markedly risen—almost threefold—over the past decade (*1*). This surge, exceeding fatal opioid overdoses in some populations, is accompanied by more prevalent cocaine use among adults; in non-Hispanic Black men and women, the death rate from cocaine overdose exceeded that from opiate overdoses (*2*, *3*). The current treatments of choice for CUD are behavioral interventions, including contingency management and cognitive-behavioral therapy (*3*). However, these treatments are not fully effective for most patients, and despite efforts to develop pharmacological agents, no medications have been proven safe and effective for the treatment of CUD (*4*). While the biological effects of repeated cocaine use have been a major focus of preclinical research in animal models (*5*–*9*), CUD remains incompletely understood and insight into the cocaine-related molecular alterations in the human brain that characterize CUD is narrow.

The mesolimbic dopaminergic system has been heavily implicated in the development of substance use disorders and addiction-related behavioral abnormalities in both clinical and preclinical studies (*10*–*16*). Research in animal models showed that cocaine and other drugs of abuse modify gene expression in the dorsal and ventral domains of the striatum, which control motivated behaviors and receive extensive input from midbrain dopamine neurons (*6*, *17*–*19*). The nucleus accumbens (NAc) of the ventral striatum primarily mediates reward, motivational salience, and reinforcement. In contrast, the caudate nucleus (CN) of the dorsal striatum primarily mediates cognition involving motor function, stimulus-response learning, and habit formation (*20*). It is hypothesized that regulation of gene expression in these brain reward and motivational centers plays a critical functional role in the persistent behavioral changes that define addiction (*6*, *21*, *22*). However, our knowledge of the maladaptive striatal gene activity that chronic cocaine use engenders in these circuits and that underlie CUD remains limited. For example, investigation of the transcriptional actions of cocaine has focused on only a fraction of the transcription factors that are likely involved (*5*), impeding a better understanding of the molecular mechanisms engaged by cocaine in development of CUD.

To address this knowledge gap, we performed RNA sequencing (RNA-seq) on both the ventral (NAc) and dorsal (CN) striatum from postmortem tissue of persons with CUD and matched control subjects. Our study uses the largest and most diverse cohort examined to date. We combined differential gene expression and network-based approaches to provide an integrative and unbiased characterization of transcriptional signatures triggered by chronic cocaine use across the striatum. Our analyses revealed a profound overlap between CUD-specific transcriptome changes in the NAc and CN, introducing the down-regulation of neuroinflammatory processes and up-regulation of synaptic transmembrane transporters and ionotropic receptors.

Using weighted coexpression analyses, we identified CUD-specific gene networks as likely drivers of the transcriptomic alterations observed in CUD, which prominently implicate synaptic remodeling downstream of cyclic adenosine monophosphate (cAMP) signaling. Because of the inherent constraints of developing a mechanistic understanding of CUD-related transcriptional activities in human striatum, it is essential to understand underlying molecular mechanisms in animal models of cocaine use. However, a key unanswered question is whether these preclinical models, in fact, reflect disease mechanisms seen in human CUD. We thus investigated whether recent preclinical findings of transcriptomic alterations in mouse models of cocaine self-administration (SA) can, in fact, recapitulate the transcriptome changes that we uncovered in persons with CUD. Our multiple-level analysis revealed robust converging pathways and evolutionarily conserved gene networks in mice and humans that characterize striatal transcriptional and physiological mechanisms in CUD.

Notably, the striatum is composed of two types of GABAergic projection neurons, medium spiny neurons (MSNs), which together constitute ~95% of the total neural population in this brain area (*19*). These distinct subtypes express either the D1 or D2 dopamine receptor and exhibit marked differences in cocaine-related neural activity and effects on drug reward: Activation of D1 MSNs in NAc promotes drug reward, whereas activation of D2 MSNs attenuates drug reward (*6*, *23*, *24*). Using MSN subtype–specific transcriptome data from mice exposed to cocaine, we illuminate the involvement of D1 versus D2 MSNs in the transcriptome alterations across conserved gene networks that are specific to CUD. Overall, this study provides a comprehensive molecular description of the transcriptional signatures associated with cocaine use in humans and highlights the validity of animal models in gaining mechanistic insight into the neuronal subtype–specific function of conserved striatal gene networks that drive molecular alterations in CUD.

## RESULTS

### Differentially expressed transcripts in the NAc and CN in CUD

We used RNA-seq to characterize the transcriptome-wide changes in gene expression within the NAc and CN of male individuals with CUD (*N* = 25) compared to well-matched control subjects (*N* = 20). Our study was balanced to include both non-Hispanic white and Black individuals (table S1), who have been shown recently to be disproportionately affected by cocaine-related overdose deaths (*2*). We used principal components analysis (PCA) to reduce the dimensionality of our dataset and to investigate whether distinct sample features could explain transcriptome variability across subject cohorts (fig. S1, A to D). This analysis showed that the sequenced samples did not segregate according to age (fig. S1B), race (fig. S1C), or postmortem interval (PMI; fig. S1D), indicating that these features did not affect transcriptome variance to a substantial degree.

**Fig. 1.**
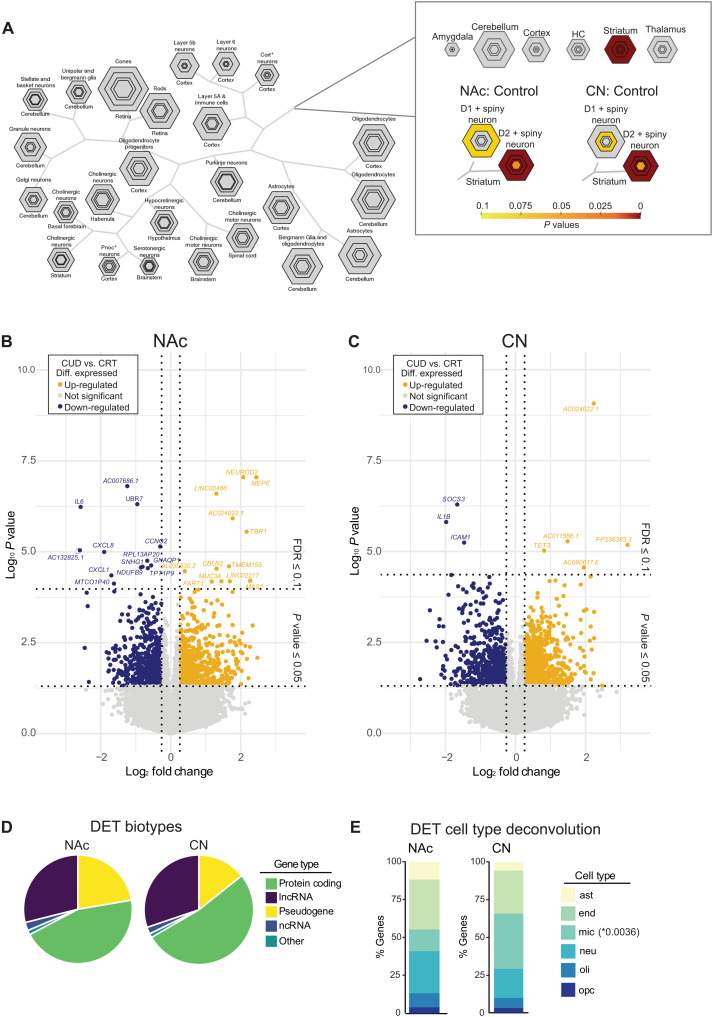
Transcriptomic changes in the NAc and CN from subjects with CUD. (**A**) Brain region SEA confirmed that NAc and CN transcriptomes were representative of the adult human striatum (*P* = 5.4 × 10^−4^, Fisher’s exact, pSI threshold of 0.0001). CSEA indicated D1 and D2 MSNs as the prevalent cellular substrates (D2 MSNs *P* = 2.8 × 10^−5^, D1 MSNs *P* = 6.7 × 10^−4^, Fisher’s exact, pSI threshold of 0.01). Hexagon rings represent gene sets of varying stringency that are specific to brain region or cell type (pSI = specificity index thresholds), with the central hexagons representing gene subsets of highest specificity. Hexagon proportions denote the gene list size. Colors indicate statistical support for enrichment of highly expressed genes at different pSI for each brain region or cell type (see bar for corresponding *P* values). Gray indicates *P* > 0.1. (**B** and **C**) Volcano plots showing differentially expressed transcripts (DETs) from CUD versus healthy control subjects in the NAc (B) and CN (C). Down-regulated genes (≤0.26 LFC (log–fold change); *P* ≤ 0.05) are represented in blue, and up-regulated genes (≥0.26 LFC; *P* ≤ 0.05) are represented in yellow. Bottom horizontal dashed line represents a nominal *P* value significance cutoff of *P* ≤ 0.05, and the top horizontal dashed line represents an FDR significance cutoff of ≤0.1; vertical dashed lines represent Log_2_FC cutoff of ±0.26 or (FC 1.2). (**D**) Biotype exploration of DETs (nominal *P* ≤ 0.05, Log_2_FC ± 0.26) in the NAc (left) and CN (right) revealed protein-coding genes represent roughly half of DETs (green; NAc: 50%, CN: 52.3%), followed by long noncoding RNAs (purple; NAc: 28.9%, CN: 30.2%). (**E**) Cell type–specific analysis of DETs in the NAc (left) and CN (right) showed a larger proportion of microglia marker DETs in the CN compared to the NAc, suggesting increased inflammatory response (Bonferroni, *P* = 0.0036). * = p < 0.05. FC, fold change; ast, astrocyte; endo, endothelial cells; mic, microglia; neu, neuron; oligo, oligodendrocytes; opc, oligodendrocyte progenitor cell; ncRNA, noncoding RNA.

In contrast, PCA identified the feature brain region as the primary source of sample variance. Thus, we subsequently included brain region as a covariate in the design of all differential gene expression analyses using DEseq2 (fig. S1A). We first confirmed that both NAc and CN transcriptomes were, in fact, representative of the adult human striatum and no other brain regions by using brain region–specific expression analysis (SEA) that was built on human data from the Brainspan collection (*P* = 5.4 × 10^−4^, Fisher’s exact,specificity index probability (pSI) threshold of 0.0001; Fig. 1A). To further identify the relevant candidate cell populations that dominate these human transcriptomes, we implemented cell type SEA (CSEA), which identified D1 and D2 MSNs as the only significantly enriched cell types, thereby confirming the known cellular components of the striatum (D2 MSNs *P* = 2.8 × 10^−5^ and D1 MSNs *P* = 6.7 × 10^−4^, Fisher’s exact, pSI threshold of 0.01; Fig. 1A).

We next investigated the impact of CUD on gene regulation in the NAc and CN. Differential expression volcano plots showed an even contribution of down-regulated (693) and up-regulated (728) transcripts in CUD in the NAc with more contribution of up-regulated transcripts in the CN [down: 640, up: 949; statistical threshold *P* ⩽ 0.05 nominal, magnitude threshold log fold change (FC) ± 0.26; false discovery rate (FDR) ⩽ 0.1 adjusted differentially expressed transcripts (DETs) indicated for NAc (25) and CN (7); Fig. 1, B and C]. Roughly half of these DETs in the NAc and CN were protein-coding genes (NAc: 50% and CN: 52.3%), with 25 to 30% representing long noncoding RNAs (lncRNAs; NAc: 28.9% and CN: 30.2%). LncRNAs represent a nontraditional but potent class of epigenetic regulators that control activity-dependent gene expression and influence several disease states (*25*). Another large category of DETs was pseudogenes (Fig. 1D), which are noncoding RNAs that were long considered inert but have been emerging with potential functions in gene silencing and in regulating mRNA stability. We next investigated whether specific cell markers for astrocytes, endothelial cells, microglia, neurons, oligodendrocytes, and oligodendrocyte precursor cells were differentially expressed in CUD using deconvolution analysis with CellDMC (*26*). Markers for the microglial compartment were significantly enriched in the CN of subjects with CUD compared to unaffected subjects (*P* = 3.7 × 10^−2^; Fig. 1E), indicating a shift in neuroinflammatory processes in CUD in this brain region (Fig. 1E).

### Transcriptional concordance between the NAc and CN converges on neuroinflammatory and retinoid receptor signaling

**Fig. 2.**
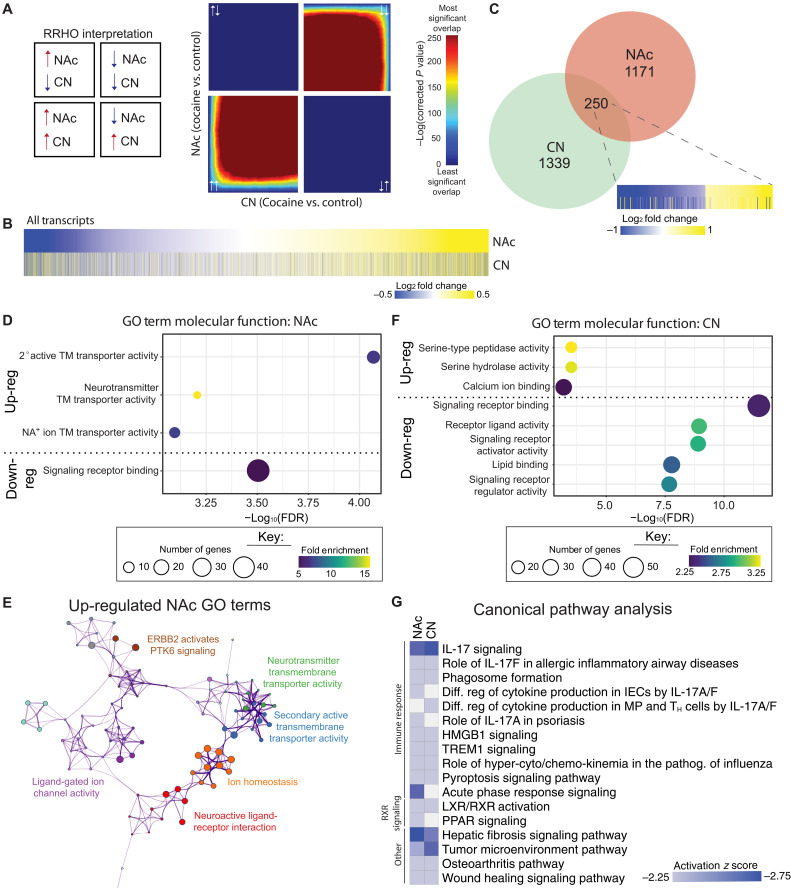
Comparison of gene expression changes between NAc and CN associated with CUD. (**A**) Threshold-free comparison of gene expression by RRHO2 analysis indicated a high degree of transcriptional overlap between cocaine-associated gene expression changes in the NAc versus CN. Pixels represent the overlap between the transcriptome of each comparison as noted, with the significance of overlap [−log_10_(*P* value) of a hypergeometric test] color coded. Bottom left quadrant includes co–up-regulated genes, top right quadrant includes co–down-regulated genes, and top left and bottom right quadrants include oppositely regulated genes (up-down and down-up, respectively). Genes along each axis are sorted from most to least significantly regulated from the middle to outer corners. (**B**) Heatmaps show all transcripts in the NAc and CN (CUD versus CTR) seeded by NAc log_2_FC. (**C**) Venn diagram of DETs between the NAc and CN with heatmap of overlapping genes (nominal *P* ≤ 0.05 and log_2_FC ± 0.26). Top molecular function GO terms enriched in (**D**) NAc DETs separated by fold change direction (up-regulated or down-regulated) feature synaptic transmission. (**E**) Network of GO terms enriched by transcripts that are up-regulated in the NAc of people with CUD compared to control subjects, featuring processes related to synaptic plasticity. (**F**) CN DETs separated by fold change direction (up-regulated or down-regulated). Size of circle represents the number of genes within the term. (**G**) Canonical pathway analysis of DETs in the NAc and CN reveals that all pathways are inactivated (negative *z* score), and most significant pathways (*q* ≤ 0.05 and ± *z* score = 2) are related to immune response. T_H_, T helper; PPAR, peroxisome proliferator–activated receptor.

Using two-sided rank-rank hypergeometric overlay (RRHO) to compare patterns and strength of transcriptional overlap in a threshold-free manner (*27*), we observed robust overlap in both up- and down-regulated gene expression between the NAc and CN (Fig. 2A). Heatmaps of differential expression of all transcripts seeded by NAc revealed largely similar patterns for expression in the NAc and CN, confirming our RRHO analysis (Fig. 2B). When we directly examined DETs in the NAc compared to the CN, we found statistically insignificant overlap using our criteria (250 overlapping genes in total; Fig. 2C), whereas heatmaps of the expression patterns of the overlapping DETs show near-identical patterns of expression. This observation underscores the value of examining these large datasets in a threshold-free manner.

To gain insight into the enrichment of DETs and their molecular functions in the two brain regions, we performed Gene Ontology (GO) analysis (ShinyGO v0.75). Given the nonsignificant overlap of DETs between the two regions, the NAc and CN were analyzed separately. Molecular function GO terms for the up-regulated DETs in the NAc were associated with transmembrane transporter activity, particularly, glutamate transporters, while down-regulated DETs were enriched in signal receptor binding as it relates to inflammatory responses, interleukin (IL) signaling, and oxidative processes (Fig. 2D and fig. S2B). Visualization of the pathway and process relationships reveals closely related clusters of up-regulated processes, i.e., transmembrane transporter and ionotropic receptor activities (Fig. 2E), and a functionally tightly linked network corresponding to down-regulated interleukin and inflammatory signaling (fig. S2A). Molecular function GO terms for the up-regulated DETs in the CN were associated with serine and calcium ion activity at the postsynaptic membrane, while the top terms for down-regulated DETs indicated reduced signaling receptor binding related to inflammatory responses and stress (Fig. 2F and fig. S2, B and C), akin to the down-regulated genes in the NAc (fig. S2B). Despite the region-specific nature of the specific up-regulated molecular function GO terms, the suppression of genes involving inflammatory responses in both brain regions was reflected in canonical pathway enrichment analysis, which revealed that most processes are related to immune response (IL-17 signaling and regulation and immune activation) and retinoid (specifically RXR) signaling (Fig. 2G).

To further examine the effects of CUD on transcriptomic differences between the NAc and CN itself, we performed differential expression analysis contrasting the NAc and CN transcriptomes in both control and CUD individuals (fig. S3, A and B). CUD is linked to an increase in differential gene regulation between these two brain regions, with a 10-fold overall expansion in the number of DETs compared to control subjects (statistical threshold *P* ⩽ 0.05, FDR = 10%, magnitude threshold logFC ± 0.26). There were no differences in the biotypes making up DETs between the control and CUD groups (fig. S3C). Furthermore, there was significant overlap (2047 DETs) between control and CUD subjects, and a heatmap of overlapping DETs showed highly similar patterns of expression in these transcripts, regulating voltage-gated ion channel activity (fig. S3, D to F).

### Distinct transcriptional regulation within the NAc between individuals with CUD versus opioid use disorder

**Fig. 3.**
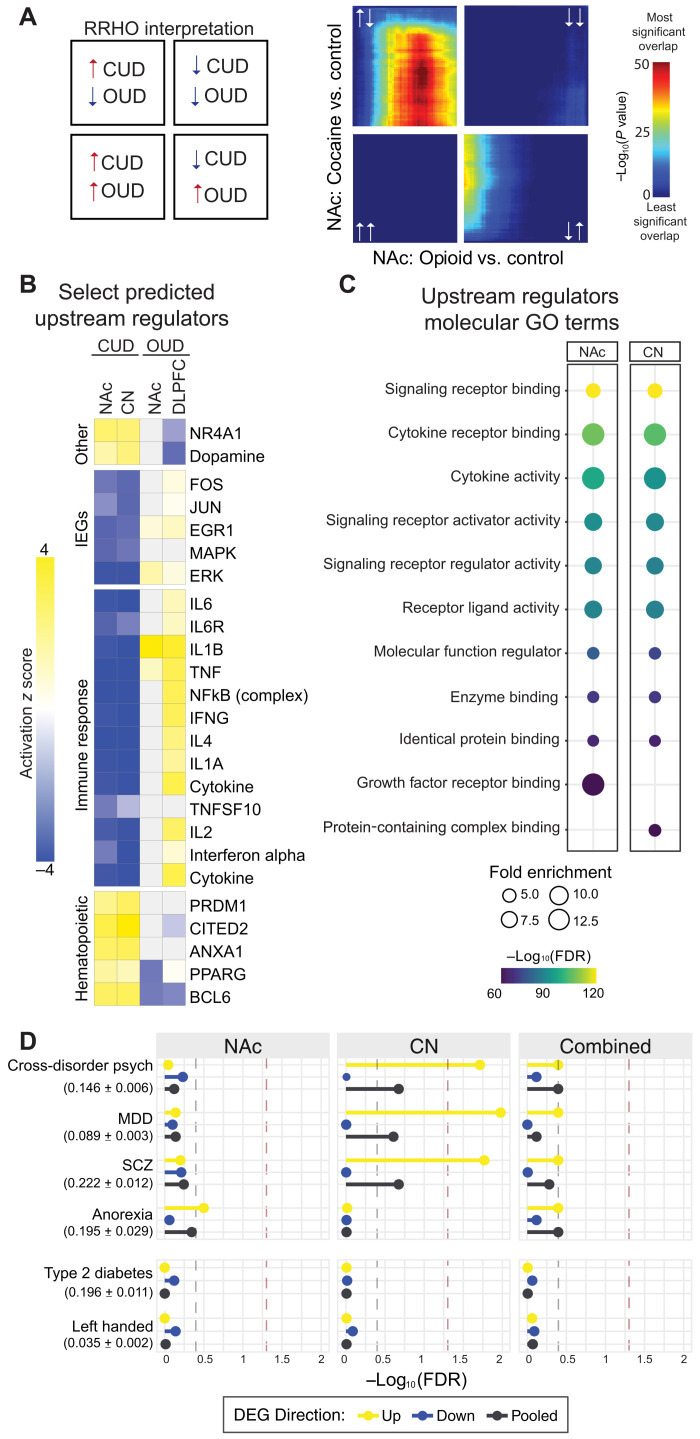
Lack of overlap of transcription changes and upstream regulators between CUD and OUD. (**A**) RRHO2 plot indicates divergent gene expression changes in NAc of individuals with CUD compared to OUD. See Fig. 2A for definition of each quadrant. (**B**) Upstream regulator analysis was conducted on DETs in CUD versus heathy control subjects in both the NAc and CN and compared to similar published analyses performed on DETs in NAc and dorsolateral prefrontal cortex from subjects with OUD (*28*). Analysis largely showed opposing patterns between the CUD and OUD datasets; select upstream regulators shown in the heatmap. Activation *z* scores in heatmaps: positive (yellow) = overrepresentation of targets activated by regulator; negative (blue) = overrepresentation of targets repressed by regulator; white = not a predicted upstream regulator. MAPK, mitogen-activated protein kinase; ERK, extracellular signal–regulated kinase. (**C**) Molecular GO term analysis of all predicted upstream regulators of DETs in the NAc and CN feature cellular signaling functions (FDR ≤ 0.05 and ± *z* score = 2; size of circle represents the number of genes within the term). (**D**) Using partitioned LD score regression, we tested common noncoding variants proximal to DETs in CN (differentially expressed (DE) *P* < 0.05), NAc (DE *P* < 0.05), or combined (DE *P* < 0.01) regions from subjects with CUD for enrichment with genetic risk variants for psychiatric traits and unrelated traits from GWASs. SNP heritability estimates are (h^2^_SNP_) displayed next to GWAS trait name (estimate ± SEM). Gray dashed line indicates nominal significance (*P* < 0.05) threshold; red dashed line indicates FDR-adjusted significance (*q* value < 0.05) threshold. MDD, major depressive disorder; SCZ, schizophrenia; ADHD, attention-deficit hyperactivity disorder.

While both cocaine and opioids have been shown to alter neurotransmitter signaling in the NAc and share some transcriptional mechanisms of gene regulation (*9*), detailed transcriptional and epigenetic changes cannot be generalized between the two drugs. To our knowledge, no studies to date have directly compared transcriptional changes in individuals with CUD and opioid use disorder (OUD). Using a recently published RNA-seq dataset from individuals with OUD (*28*), we examined substance-specific transcriptional differences in NAc tissue. RRHO revealed substantial discordant regulation of up- and down-regulated transcripts (Fig. 3A). Given the opposing transcriptional regulation between cocaine- and opioid-related gene expression revealed by threshold-free transcriptomic analysis, we also examined whether there are distinct predicted upstream regulators of DETs in both studies. While there were several overlapping upstream regulators, such as EGR1 (Early Growth Response 1) and NR4A1 (Nuclear Receptor Subfamily 4 Group A Member 1), many showed opposite activation. For example, both EGR1 and the inflammatory factor tumor necrosis factor–α (TNFα) are predicted upstream regulators in individuals with CUD or OUD; however, they are predicted to be inactive in CUD but activated in OUD (Fig. 3B). Furthermore, inflammatory cytokines and signaling factors such as nuclear factor κB (NFκB) are among the top predicted upstream regulators of DETs in OUD (*28*) yet showed opposite regulation in CUD (Fig. 3B). Molecular function GO term analysis of all predicted upstream regulators again point to immune function and regulation (Fig. 3C).

### Differential gene expression implicates genetic risk factors for substance use–related traits

Cocaine-induced gene expression abnormalities have been linked to neuronal and circuit adaptations (*6*) and thus may directly point to genetic risk factors that contribute to CUD. Genetic risk variants for CUD have been investigated by a small number of genome-wide association studies (GWASs) (*29*), which estimate the heritability for cocaine dependence using single-nucleotide polymorphisms (SNPs) to be around *h*^2^_SNP_ ~ 0.3 (*30*–*33*). To assess likely targets, we overlapped DETs in NAc and CN from our study with gene variants linked to CUD in recent GWASs (*30*–*32*). Despite the limitations presented by the highly polygenic nature of psychiatric disorders and the statistical price paid of testing millions of genetic variants in GWASs, we uncovered several genes of potential interest (table S2), including *NR4A1*, an important regulator of cocaine-induced gene regulation that plays an integral role in neuronal homeostasis and that has been linked to cocaine-evoked behaviors (*17*, *34*, *35*). These risk variants could show differential expression before cocaine use or exhibit altered drug responses that affect neural circuit adaptations, increasing the susceptibility to CUD.

We further explored whether common single-nucleotide variants (SNVs) located near CUD-related DETs were enriched for genetic risk variants linked to substance use–related traits and psychiatric disorders using partitioned linkage disequilibrium score regression (*36*). These genetic risk loci primarily reside in noncoding regions of the genome and are thought to serve as cis-acting regulators of nearby gene expression. Our analyses revealed significant enrichment of SNVs near up-regulated transcripts in the CN of CUD subjects for genes associated with major depressive disorder (MDD) and schizophrenia (Fig. 3D). Risk taking and impulsivity are strongly linked to CUD and other substance use disorders (*29*). We found that SNVs near up-regulated DETs in CUD enrich for risk loci linked to risky behavior by GWASs (Fig. 3D), revealing that substance use–related genetic risk factors are significantly linked to CUD-related transcriptome changes in the CN but not the NAc. These findings further indicate that the relationship between NAc and CN transcriptomes is important to the expression of genetic risk, not just region-specific gene regulation. In contrast, as would be expected, GWAS traits for left-handedness and type 2 diabetes (T2D) were unrelated to NAc and CN DETs in CUD (Fig. 3D).

### Threshold-free genome-wide expression overlap between human CUD and mouse cocaine self-administration

**Fig. 4.**
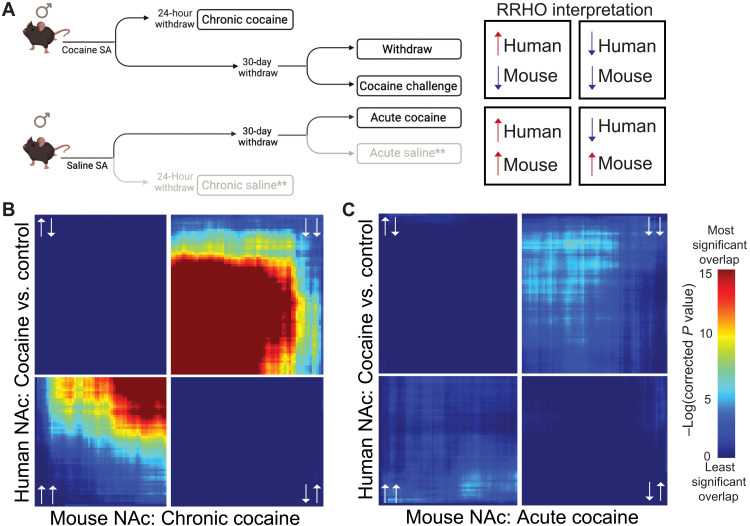
Transcriptional changes in human CUD brain regions overlap with chronic but not acute cocaine exposure in mouse self-administration models. (**A**) Schematic outlines the experimental design used for the mouse cocaine self-administration study and indicates the cohorts used for comparative transcriptome analyses. Male C57BL/6J mice self-administered either cocaine or saline control for 10 consecutive days. One cocaine cohort was euthanized 24 hours after its last cocaine self-administration (chronic cocaine), whereas another cohort of animals was group housed in their home cage for 30 days before they were given saline (withdrawal) or cocaine (cocaine challenge). A cohort of drug-naïve mice that self-administered saline was given cocaine to model first-time drug experience (acute cocaine). See Materials and Methods and GSE110344 on GEO (*17*) for additional experimental detail. ** indicates control conditions(**B**) RRHO2 analysis was used to examine threshold-free overlap in gene expression in the NAc of genes conserved between our human CUD cohort and mice that self-administered cocaine (*17*). The transcriptome of mice that were euthanized without prolonged withdrawal after the last self-administration session (chronic cocaine) showed significantly concordant gene expression changes in NAc when compared to human CUD-related transcriptomes. (**C**) Drug-naïve mice exposed to acute cocaine (acute cocaine) showed only minor overlap in gene expression changes within the NAc compared to CUD. The different patterns of overlap found with 30 days of cocaine withdrawal are shown in fig. S4. See Fig. 2A for definition of each quadrant of RRHO2 plots.

While animal models have been a major focus of research to better understand the molecular pathophysiology of addiction, it remains uncertain whether the cocaine-related molecular alterations explored in rodent models mirror transcriptomic changes in humans with CUD. To assess the ability of cocaine self-administration in mice to mirror transcriptional changes in individuals with CUD, we conducted a direct comparison of the human CUD transcriptomic patterns and those in a mouse cocaine self-administration study where transcriptional regulation was assessed in the NAc using RNA-seq (Fig. 4A) (*17*). In this earlier study, male mice voluntarily consumed cocaine (~10 mg/kg) per day for 10 consecutive days with a saline self-administration control cohort run in parallel. Using RRHO, we found high concordance in gene expression patterns between animals self-administering chronic cocaine and the human subjects with CUD (Fig. 4B). In contrast, acute cocaine in drug-naïve mice showed no significant overlap of transcriptome regulation (Fig. 4C), indicating that a history of chronic drug exposure causes molecular changes that are unique and better model CUD-related gene expression abnormalities in humans. We conducted the same analysis for mice euthanized after 30 days of withdrawal from cocaine self-administration and found no significant overlap (fig. S4A). Thirty days of withdrawal followed by an acute cocaine challenge in mice showed significantly opposing transcriptomic regulation compared to individuals with CUD (fig. S4B). It will be important in future studies to obtain brain tissue from humans with CUD who die after periods of prolonged withdrawal without and with relapse to test whether transcriptional patterns at these time points also converge with observations in mouse models.

The finding that the human transcriptional abnormalities most closely resembled those seen in mice shortly after chronic cocaine self-administration is consistent with the fact that most individuals included in our cohort were active drug users at the time of death. By comparing the transcriptional landscapes of self-administering mice and humans with CUD, these data establish the ability of mouse models to reflect disease mechanisms and provide strong support for the validity of reverse and forward translation approaches.

### Conserved gene networks in CUD and mouse cocaine self-administration implicate synaptic remodeling downstream of cAMP

**Fig. 5.**
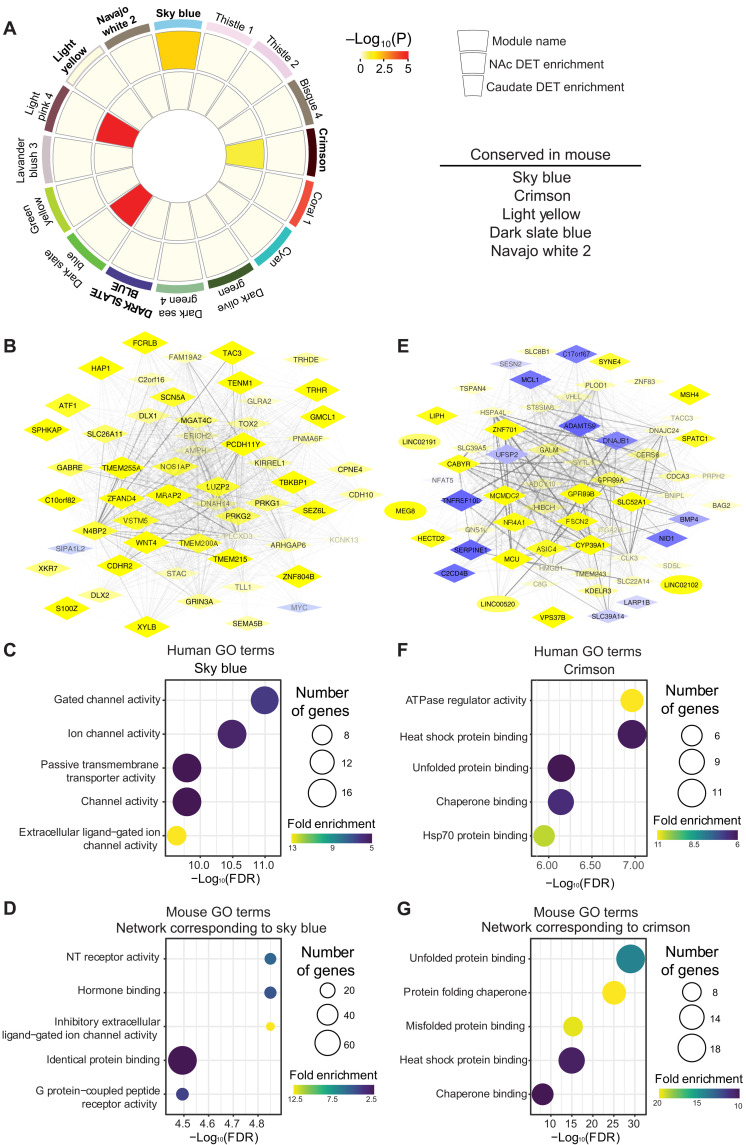
Identification of coexpression gene networks conserved between human CUD and mouse cocaine self-administration. (**A**) Circos plot for the top CUD-specific 16 modules identified by WGCNA on human transcriptomes (MDC score > 2.5). Module names (an arbitrary color) are indicated on the outside of the Circos plot. Enrichment for either NAc or CN DETs are indicated by the inner rings of each module, with increasing warm colors indicating increasing log_10_(*P* value). WGCNA was conducted on published mouse self-administration transcriptome data from NAc and CN (*17*) to detect modules that are conserved across species. (**B**) Arachne plot shows gene expression network of the human skyblue module. Molecular function GO terms significantly enriched in the (**C**) human skyblue network and its corresponding mouse module, (**D**) mouse green yellow. (**E**) Arachne plot shows the gene expression network of the human crimson module (diamond shape signifies protein coding genes, and ellipse signifies lncRNAs). Molecular function GO terms significantly enriched in the **(F)** human crimson network and its corresponding mouse module, (**G**) mouse light cyan. Circle size indicates number of genes in the molecular function term. Heatmap indicates the fold enrichment score (higher enrichment in purple and low enrichment shown in yellow) with FDR ≤ 0.05. NT, neurotransmitter; ATPase, adenosine triphosphatase.

To generate more functional insight into CUD-related genes, we used weighted gene coexpression network analysis (WGCNA), which generates transcriptional networks based on the correlated expression of genes across a population of samples (*37*, *38*). Network modules were generated by collapsing across the NAc and CN to increase power, an approach supported by the substantial transcriptional overlap between these striatal brain regions in CUD. This analysis identified 63 coexpression modules with strong evidence for preservation in the striatum (Zsummary value > 10). Module differential connectivity (MDC) scoring was used to detect 16 CUD-specific modules that showed considerably more coordinated expression of transcripts, i.e., significantly higher connectivity, in subjects with CUD compared to control subjects (MDC score > 2.5, FDR < 0.05; Fig. 5A and table S3). To gain insight into the biological function of key CUD-specific modules, we first focused our analysis on networks that showed enrichment of DETs in either NAc or CN (Fig. 5A). One major network that was of particular interest with greatly increased connectivity in CUD was the green module (arbitrary color name; fig. S5A), which also showed significant enrichment of DETs in the CN. Ingenuity Pathway Analysis (IPA) confirmed that genes within the green module are predicted to be controlled by dopamine and cocaine (fig. S6, A and B). It further revealed several upstream transcriptional activators, including CREB1, E2F1, SMAD3, AP1, and SMARCA4 (fig. S5B), all of which have been implicated recently in addiction-like behaviors in mice that self-administer cocaine (*11*, *17*, *39*, *40*). In contrast, the TWIST2 transcriptional regulator—which controls the inflammatory cytokines *IL-6, IL-1*β, and *TNF*α (*41*)—was predicted to be inhibited in the CUD-related regulation of this module (fig. S5B).

We again wanted to leverage the integration of human subjects with CUD to mouse self-administration models. Thus, we investigated whether the human CUD-specific modules are conserved in the widely used mouse model of cocaine self-administration. We conducted a direct comparison of the CUD-specific human gene networks and performed WGCNA on a recently published comprehensive NAc transcriptome data from the mouse cocaine self-administration mentioned above (*17*). Using WGCNA, we identified 61 coexpression modules in the mouse NAc, of which 12 networks showed greatly increased connectivity in animals with a history of chronic cocaine intake compared to drug-naïve control mice (MDC score > 2.5, FDR < 0.05; table S4). We estimated cross-species module conservation by comparing module membership between the human and mouse cohorts. We identified 29 human modules that were conserved in the mouse (odds ratio > 3.5, Bonferroni *P* < 0.05; table S5), of which five networks were characterized by high differential connectivity in CUD (MDC score > 2.5, Bonferroni *P* < 0.05; Fig. 5A and table S6). One such prominent and highly conserved module was the skyblue module (conservation Bonferroni *P* = 1.89 × 10^−4^), a network that showed enrichment for DETs in the human NAc and differential tissue correlation of its CUD-related gene responses (median NAc > CN, *P* = 1.42 × 10^−5;^ Fig. 5, B and C). IPA revealed that the top active canonical pathways in the skyblue network involve glutamate receptor signaling (*z* score = 2, *P* = 6.54 × 10^−6^), synaptogenesis signaling (*z* score = 2.3, *P* = 3.85 × 10^−4^), and dopamine-DARPP32 feedback in cAMP signaling (*z* score = 2.2, *P* = 1.78 × 10^−3^). DARPP32 is a well-known phosphoprotein in dopaminoceptive neurons that modulates electrophysiological, transcriptional, and behavioral responses to cocaine and other drugs of abuse (*42*–*44*). Another human module that was conserved as a cocaine-regulated network in the mouse NAc was the crimson module. Upstream regulator analyses predicted that this network is regulated by activation of EP400, a key component of chromatin remodeling complexes that generally stimulates transcription, and EP2 (or PTGER2; *z* score = 2, *P* = 2.25 × 10^−3^), the striatal receptor for prostaglandin E2 that is produced in response to D1 and D2 dopamine receptor stimulation (*45*), and that was shown to be up-regulated in the NAc of rhesus macaques with long term cocaine self-administration (*46*). On the other hand, CUD-related inhibition was predicted for the upstream regulation of this module by interferon-γ cytokine signaling (*z* score = −2.1, *P* = 4.93 × 10^−3^).

To investigate and further characterize the molecular processes controlled by these conserved networks, we performed GO enrichment analyses on both the human skyblue and crimson modules, as well as on the corresponding mouse modules (mouse greenyellow and mouse lightcyan, respectively; table S6). The human skyblue network was enriched in components of synaptic membranes that regulate the activity of ionotropic receptors and transmembrane transporters (Fig. 5C), among them are potassium channels (e.g.*, KCNC2*, *KCNC4*, and *KCNK13*) and glutamate receptor subunits (e.g., *GRIA4* and *GRIN3A*) that have been previously implicated in the behavioral manifestation of addiction (*47*–*51*). Notably, the GO terms enriched in the corresponding mouse module (Fig. 5D) also included many of these same key gene products found in the human module, e.g., *GRIN3a*, *GABRE*, and members of the glycine receptor family (*GLRA1* to *GLRA3*), which have also been shown to be regulated in the NAc of rhesus macaques with long-term cocaine self-administration (*46*). Likewise, the predicted molecular function of the crimson network in humans and the corresponding mouse module overlapped and enriched for identical GO categories (Fig. 5, E to G), implicating stress response pathways and chaperone complexes in CUD. Key members of the nuclear receptor (NR) family emerged as hub genes across prominent human modules, including *NR4A1* (green), *NR4A2* (thistle), and *NR4A3* (crimson). These transcription factors have been identified as critical for CREB-mediated gene responses in rodent models of cocaine self-administration and are hypothesized to interact with CREB to regulate different transcriptional programs in a context-specific manner (*11*, *17*, *34*, *52*–*54*). We found CREB1—heretofore heavily implicated in cocaine action in the NAc in rodent models (*1*)—to be a predicted upstream regulator in every one of these functionally distinct modules (green *z* score = 0.9, *P* = 1.05 × 10^−4^; thistle *z* score = −0.7, *P* = 3.89 × 10^−8^; crimson *z* score = 0.5, *P* = 2.19 × 10^−2^).

### D1 and D2 MSN-specific cocaine regulation of conserved gene regulation in humans and mice

To gain insight into the potential involvement of D1 and D2 MSNs in the CUD-specific gene modules that were conserved in cocaine self-administering mice (see Fig. 5), we used a recently published dataset that examined the effects of cocaine on NAc transcription in a cell type–specific manner (*55*). D1 and D2 MSN transcriptomes were generated from NAc in two transgenic mouse lines (*Drd1a::Egfp-L10a* or *Drd2a::Egfp-L10a*) that enabled fluorescent-activated nuclei sorting for either cell type combined with population-specific RNA-seq. The following groups were included in this mouse study: effects of acute cocaine (1 hour) in drug-naïve mice, effects of prolonged withdrawal (30 days) from chronic cocaine, and effects of an acute cocaine challenge (1 hour) after prolonged withdrawal from chronic cocaine, all compared to saline-treated control animals.

**Fig. 6.**
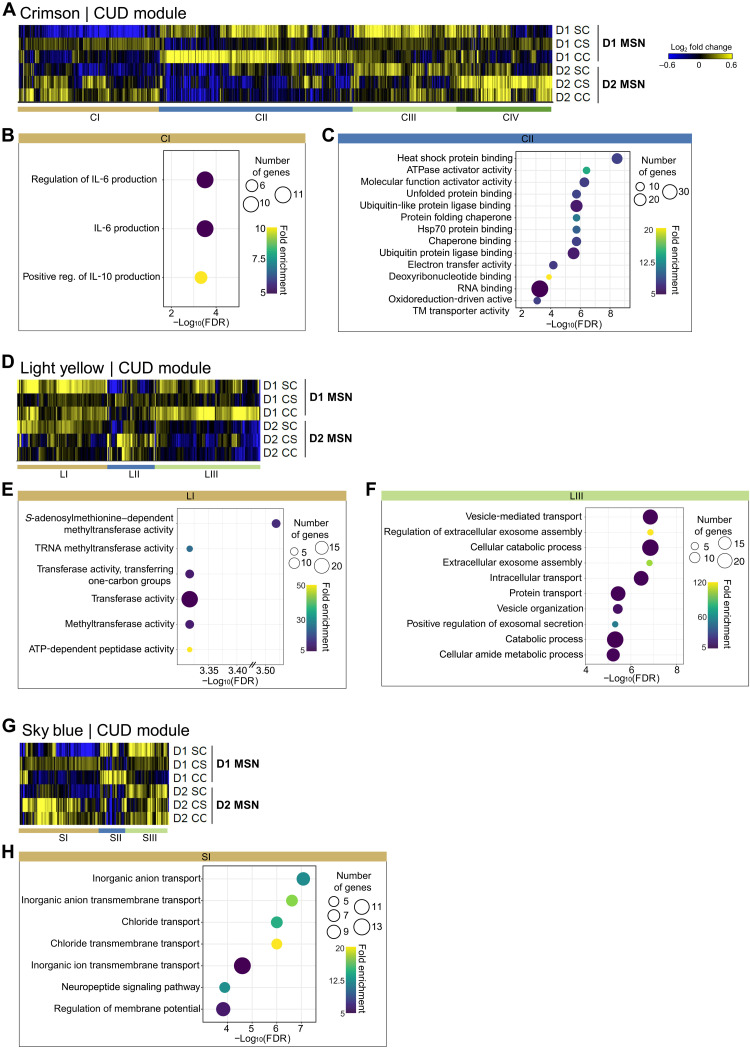
Expression patterns of conserved human CUD gene networks in mouse D1 and D2 MSNs. To examine the contribution of D1 versus D2 MSNs in cocaine-related transcriptional regulation of conserved human (**A** to **C**) crimson, (**D** to **F**) lightyellow, and (**G** to **H**) skyblue networks, we used a recently published dataset that characterized cocaine-related changes in subtype-specific gene expression in the NAc (*55*). In this dataset, animals received a cocaine (20 mg/kg injection i.p.) to profile and compare the transcriptional response in D1 versus D2 MSNs to acute cocaine in drug-naïve mice (SC, saline-cocaine) with that to a cocaine challenge in withdrawal animals (cocaine challenge, CC; 30-day withdrawal following 10 days of chronic cocaine) and to a saline challenge in withdrawal animals (CS, socaine-saline) when compared to control saline animals (SS, saline-saline). GO term analysis was performed on major gene clusters (κ-means clustering, one minus Pearson correlation) within the respective conserved modules for crimson (B and C), lightyellow (E and F), and skyblue (H). ATP, adenosine 5′-triphosphate.

We first evaluated cell type–specific transcriptional responses in the conserved human crimson module (Fig. 6, A to C) using κ-means clustering to partition transcripts into subgroups according to their common regulation across the mouse cohorts. One prominent gene group that was up-regulated in D1 MSNs—but not D2 MSNs—following an acute cocaine dose either in drug-naïve mice or in mice after prolonged withdrawal from chronic cocaine (i.e., cocaine relapse) is cluster II. GO enrichment analysis showed that cluster II participates in cellular stress responses, indicating that this primary molecular function that was predicted for the crimson module in CUD is specific to D1 MSNs (Fig. 6C). Cluster I within this conserved network of regulated genes, which controls the production of IL-6, was suppressed by acute cocaine and by cocaine relapse in D1 MSNs (Fig. 6B). *Il-6* is one of the significantly down-regulated genes in the NAc of subjects with CUD, indicating that D1 MSNs partially drive the transcriptional response within this module.

In the conserved human lightyellow module, most of genes were primarily induced in D1 MSNs—but not in D2 MSNs—by acute cocaine and cocaine relapse (Fig. 6D). This conserved gene network that comprised epigenetic enzymes with methyltransferase activity (Cluster I) and genes that positively regulate vesicle-mediated transport, exosome assembly, and exosome secretion (Fig. 6, E and F). In contrast, the largest cluster within the conserved human skyblue module was up-regulated after prolonged withdrawal from chronic cocaine in both D1 and D2 MSNs and highly expressed in D2 MSNs upon cocaine relapse (Fig. 6G). GO analysis showed that this cluster participates in postsynaptic membrane regulation and encodes transmembrane transporters and ionotropic channels referenced above, such as glycinergic and γ-aminobutyric acid receptor complexes (Fig. 6H).

## DISCUSSION

The results of our study map transcriptome-wide changes in gene expression in two striatal regions of humans with CUD. We identify cocaine-related gene regulation as well as highly regulated gene networks in NAc and CN that are most prominently affected by this syndrome. While these two brain regions exhibit distinct transcriptomes and CUD-related changes in gene expression, we identified a subset of DETs that is regulated similarly by CUD. Our findings further support the hypothesis that cocaine-responsive genes participate in the vulnerability to substance use disorders, as we found a convergence of up-regulated DETs in individuals with CUD and genes associated with risk-taking behavior. Likewise, we found that genes linked to schizophrenia and MDD are dysregulated in CUD, indicating that these two complex disorders recruit some of the same gene regulatory and neural circuit systems. In addition, our study reveals major differences in transcriptional abnormalities within the NAc associated with CUD versus OUD based on published datasets for the latter (*28*).

We establish that large portions of the molecular pathology in the striatum that underlies CUD in humans can be recapitulated in mice that self-administer cocaine, thereby providing important validation for the use of mouse models to study the pathophysiological basis of CUD. Analyses in mice especially implicate D1 MSNs in the gene expression abnormalities seen in humans with CUD. Last, it is important to emphasize that our human cohort includes a significant number of Black individuals, who have not been well represented in prior transcriptional studies of CUD, despite the recent evidence that such individuals are especially vulnerable to cocaine-related overdose deaths (*4*). Together, these findings represent a considerable advance in our understanding of the molecular abnormalities in CUD and provide a highly valuable resource for future investigations.

One key finding from this study is that neuroinflammatory processes are suppressed in the striatum of people with CUD. These results demonstrate directionally opposite regulation of key cytokines compared to recent work on OUD (*28*). Although chronic exposure to drugs of abuse is typically associated with the release of proinflammatory cytokines (*16*), cocaine dependence and acute cocaine exposure have been shown to reduce levels of the proinflammatory cytokine, IL-6 (*56*, *57*). In our study, we found that this factor was significantly down-regulated in both NAc and CN transcriptomes in individuals with CUD, concomitant with changes in microglia markers that are indicative of suppressed neuroinflammation. Canonical pathway analysis further revealed down-regulation of IL-17 signaling as a key upstream regulator of the transcriptomic changes in NAc and CN. IL-17 signaling promotes microglial activation and neuroinflammation (*58*), including up-regulation of *IL-6* and *TNF*α, thus indicating that acute cocaine suppresses neuroinflammatory responses in CUD. These findings mirror studies that have found differing effects of chronic cocaine and opioids on the hypothalamic-pituitary-adrenal (HPA) axis (*59*). Stress and elevated levels of corticotropin-releasing hormone (CRH) were found to promote drug craving and relapse in cocaine withdrawal (*59*, *60*). In contrast, opioid withdrawal was found to decrease the response of the pituitary to CRH (*61*). However, chronic cocaine exposure has been linked to tolerance of the HPA axis to the stimulatory effects of cocaine in rats (*62*), a condition that is more reflective of the CUD subjects characterized here. Our findings on opposing neuroinflammatory responses to cocaine and opioids thus add to the growing literature supporting distinct impact on physiological systems involved in the drug response. However, while our comparative analyses improve our understanding of the transcriptional dysregulation in these disorders, the OUD study included female individuals and largely encompassed white individuals. Additional molecular studies in rodent models of OUD and CUD that integrate measures of stress response and immune function should greatly advance our mechanistic understanding of the gene-regulatory dichotomy between these two disorders.

Concordant patterns of gene regulation and deduced biological processes in both NAc and CN showed a significant similarity in the CUD-related transcriptome changes between these two brain regions, which involved up-regulation of numerous ionotropic receptors and genes linked to postsynaptic membrane signaling. Our WGCNA approach to investigating gene networks in both human CUD and mouse cocaine self-administration identified several key modules that are highly conserved. One major conserved gene network was the crimson module, which participates in stress response pathways and chaperone complexes in CUD. Cocaine is well known to increase reactive oxygen species and oxidative stress in striatal regions, which is further exacerbated by excessive dopamine levels and increased glutamate release in CUD (*63*). Integrating the human transcriptomes with our D1 and D2 MSN transcriptome data from mice (*55*) revealed the potential involvement of both cell types in the regulation of conserved gene networks identified by WGCNA. However, our cross-species analyses provided particular evidence for the implication of D1 MSNs in CUD. For example, regulation of the conserved human crimson network is driven in D1 MSNs, which revealed down-regulation of genes controlling IL-6 production and up-regulation of genes engaged in stress responses in NAc of mice after acute cocaine or cocaine relapse. This analysis is particularly useful because it is currently not technically possible to obtain high-resolution differential transcriptomic mapping for D1 versus D2 MSNs from human striatum. While single-nucleus sequencing can successfully identify cellular subtypes, direct combination of arbitrary clustering and differential testing tools is more limited, with further constraints markedly restricting the detection of lowly expressed genes. Future evolution of single-cell and spatial sequencing technologies holds promise for more direct insight into subtype-specific transcriptome alterations in human CUD.

Our analysis identified numerous transcription factors that are predicted to be upstream of the gene expression abnormalities associated with CUD. Most are shared between the NAc and CN. Notably, several of these predicted upstream transcription factors have been implicated previously in cocaine action in rodent models, in particular, AP-1 (FOS/JUN) family, EGR family, NFκB, E2F, and several nuclear hormone receptors (*5*, *11*). At the same time, several additional transcriptional regulators not previously implicated were identified in this study, which now warrant direct investigation of their mechanistic roles in cocaine addiction. Notably, as our integrative cross-species analyses focused on large datasets from male humans and mice, future studies on human females will be of utmost importance given sex differences observed in animal models.

The use of animal models has increased our understanding of how cocaine and other addictive drugs induce transcriptomic plasticity in addiction-relevant brain regions, first and foremost in the striatum (*1*–*3*). However, while self-administration procedures have firmly established that drugs of abuse, including cocaine, function as reinforcers in animal models, it has remained unclear whether the cocaine-related molecular alterations explored in rodent models sufficiently reflect transcriptomic changes in people with CUD. Our study highlights the validity and value of preclinical animal models to provide crucial insight and mechanistic information across transcriptional networks, biological pathways, and even neuronal cell types in striatum that result in the detrimental neuroadaptations of CUD. One must carefully consider treatment paradigms and experimental endpoints in these animal models to capture the distinct molecular features of CUD, because chronic drug use will present differently at the transcriptional level based on early versus late withdrawal times or on relapse. Ultimately, information gathered from animal models will be essential for leveraging an improved understanding of the molecular pathology of CUD for the development of therapeutics for this syndrome.

## MATERIALS AND METHODS

### Postmortem brain samples

Biospecimens were provided by the University of Miami Brain Endowment Bank, an Institutional Review Board–approved biorepository that holds frozen brain tissue annotated with demographic information and clinical variables. Postmortem male brain specimens were obtained from unaffected control subjects and from cocaine-related deaths that came to routine autopsy. Medicolegal investigations and certifications of the cocaine intoxication deaths were conducted by forensic pathologists to determine the cause and manner of death. The circumstances of death and blood and brain toxicology were reviewed to identify sudden deaths due to the toxic effects of chronic cocaine abuse in persons who met the criteria for CUD (*n* = 25). Drug-free age-matched control subjects (*n* = 20) were selected from homicides, accidental, or natural deaths that had negative urine screens for all common drugs in decedents with no history of licit or illicit drug use before death.

The brain samples were matched for sex (male), age, race, smoking history, PMI, and brain pH to normalize factors that can affect brain RNA quality. RNA integrity numbers did not significantly differ between cases and controls (table S1). This study used male samples only because of the preponderance of males in the available brain bank. Cocaine use still predominates in males overall and the incidence of cocaine intoxication deaths follows this pattern. In addition, the rates of polydrug abuse and psychiatric comorbidity are higher among females, further excluding females based on the criteria for subject inclusion for the present study.

### Total RNA-seq and differential gene expression analyses

To isolate RNA from the samples in TRIzol LS, we used the Zymo Directzol RNA Miniprep Kit (Zymo Research, R2050). We performed deoxyribonuclease treatment on all samples to ensure the removal of genomic DNA before proceeding with library preparation. RNA was analyzed for size distribution and quantity using Bioanalyzer 2000 (Agilent Technologies, USA). We used the Takara SMARTer Stranded Total RNA-seq Library Preparation Kit to create indexed libraries from ribo-depleted RNA from each sample for sequencing (Takara Bio, 634839). All libraries were generated simultaneously using a multiplexed library preparation strategy to ensure no variation in processing due to batch effects. Resulting libraries were analyzed for size distribution and quantity using the Agilent Bioanalyzer and TapeStation (Life Technologies) before sequencing. Molar equivalent libraries were pooled for multiplexed sequencing on the Illumina NovoSeq. We performed 2× 150–base paired-end RNA-seq and obtained a total average of 60,000,000 reads per sample. The number of independent tissue samples included in the final analysis was between 20 and 25 individuals per group. The raw FASTQ sequencing data will be made publicly available in the Gene Expression Omnibus (GEO) with accession no. GSE214267.

After filtering, pair-wise differential expression comparisons using DEseq2 were performed to identify DETs in comparisons of control individuals versus individuals with CUD in the NAc and the CN (*64*). Transcripts with a nominal *P* ≤ 0.05 and a log_2_FC ± 0.26 (i.e., FC ± 1.2 or 20% expression change) were considered differentially expressed. Chi-square test was used to examine differences in the proportion of DET biotypes between NAc and CN.

### GO and predicted upstream regulator analysis

Functional annotation for gene set enrichment analysis (GO) of molecular functions was performed using ShinyGO 0.76 with gene identities of DETs (nominal *P* ≤ 0.05), with an FDR ≤ 0.05 cutoff for significantly enriched GO terms (*65*). IPA (Qiagen) was used to predict upstream regulators and significant pathways of DE transcripts. These determinations were based on the log FC of DETs in CUD compared to control subjects. GO networks were visualized using Metascape (*66*). Biotypes for transcripts with annotations were compared between DE transcripts in NAc and CN of subjects with CUD. Predicted upstream regulators and molecular pathways were identified with the use of IPA software (Qiagen Inc.) (*67*). These determinations were based on the log FC of DEGs in resilient or susceptible animals compared with controls.

### Rank-rank hypergeometric overlap

Threshold-free genome-wide transcriptomic overlap analysis was conducted using RRHO2 (*27*). Briefly, the RRHO2 ranks ENSEMBL gene lists by a signed *P* value, that is, the –log_10_(*P* value) multiplied by the sign of the FC and generates matrices of overlapped genes between RNA-seq lists of interest independent of whether individual RNAs are differentially expressed or not. RRHO was used to evaluate the overall patterns of gene expression between the NAc and CN. For comparisons of human CUD NAc and mouse self-administration NAc transcriptomes, DESeq2 was used to obtain differentially expressed genes in mouse self-administration, comparing the groups SC versus SS, CS versus SS, and CC versus SS using the datasets GSE110344 on GEO (*17*). Conserved ENSEMBL genes were assessed. Only transcripts that remained following filtering were kept, and axes were ranked as detailed above.

### Weighted gene coexpression analysis

WGCNA was used to identify coexpression networks across all human subjects (CUD and healthy controls combined). Gene expression data for all brain regions were first compiled and first covariate corrected for age, race, and PMI. The genes in the bottom quartile of variance and expression were removed. Samples with a cross-sample correlation greater than two SDs from the mean were removed. Linear relationships between gene expression were estimated by pairwise Pearson correlation. An adjacency matrix was calculated from this correlation matrix by raising it to a positive power, β, which was determined by a “scale-free” power law connectivity distribution (*38*). The adjacency matrix was then quadratically transformed to a topological overlap matrix (*68*) before being grouped into modules by a hierarchical clustering algorithm (*37*). Each module was named with an arbitrary color, and “gray” was used to identify genes that do not segregate into any particular module. Module robustness was determined empirically by repeatedly splitting the gene expression data in training and test sets and calculating a module preservation score between each network (*69*). A composite preservation statistic (*Z*) and empirical *P* values were calculated, and a previously characterized threshold (*Z* > 10) and Bonferroni *P* value threshold (< 0.05) were used to assess for preservation. A similar protocol was used to separately analyze data from a previously published dataset of male mouse NAc self-administration. MDC was used to quantify differences in coexpression between CUD and controls. MDC is the ratio of the average of network connectivity between cases and controls for genes in a particular module. MDC of 2 indicates that the average correlation of genes in a module are twice as strong in cases compared to controls. MDC >1 suggests gained connectivity while MDC < 1 indicates loss of connectivity. Two separate FDR estimate were calculated by randomly shuffling samples and genes, and the final FDR was determined by selecting the larger estimate. A module was considered differentially connected is FDR < 0.01 and MDC > 2.5 or < 0.75. We used Cytoscape software platform to visually reconstruct Arachne plots (*70*). Fisher’s exact tests were conducted using the super exact test package in R as described previously to determine the enrichment of DETs in the NAc and CN (*71*). For select CUD-related gene modules that were preserved in mouse self-administration, we performed D1 and D2 MSN-specific transcriptome analysis on conserved ensemble genes using subtype-specific RNA-seq datasets from mice that experienced chronic cocaine experience and prolonged withdrawal, with or without cocaine challenge (*55*).

## Notes

View/request a protocol for this paper from *Bio-protocol*.
