# Supplementary material for: Convergent abnormalities in striatal gene networks in human cocaine use disorder and mouse cocaine administration models
Source: Sci Adv. 2023 Feb 10;9(6):eadd8946. doi: 10.1126/sciadv.add8946 (PMC9916993; doi:10.1126/sciadv.add8946)
Supplement: Supplementary file 1 — Figs. S1 to S6 [file sciadv.add8946_sm.pdf]

Science Advances
AAAS

Supplementary Materials for

Convergent abnormalities in striatal gene networks in human cocaine use disorder and mouse cocaine administration models

Philipp Mews *et al.*

Corresponding author: eric.nestler@mssm.edu; Philipp Mews, philipp.mews@mssm.edu

Sci. Adv. **9**, eadd8946 (2023)
DOI: 10.1126/sciadv.add8946

This PDF file includes:

Figs. S1 to S6
Legends for tables S1 to S6

Other Supplementary Material for this manuscript includes the following:

Tables S1 to S6

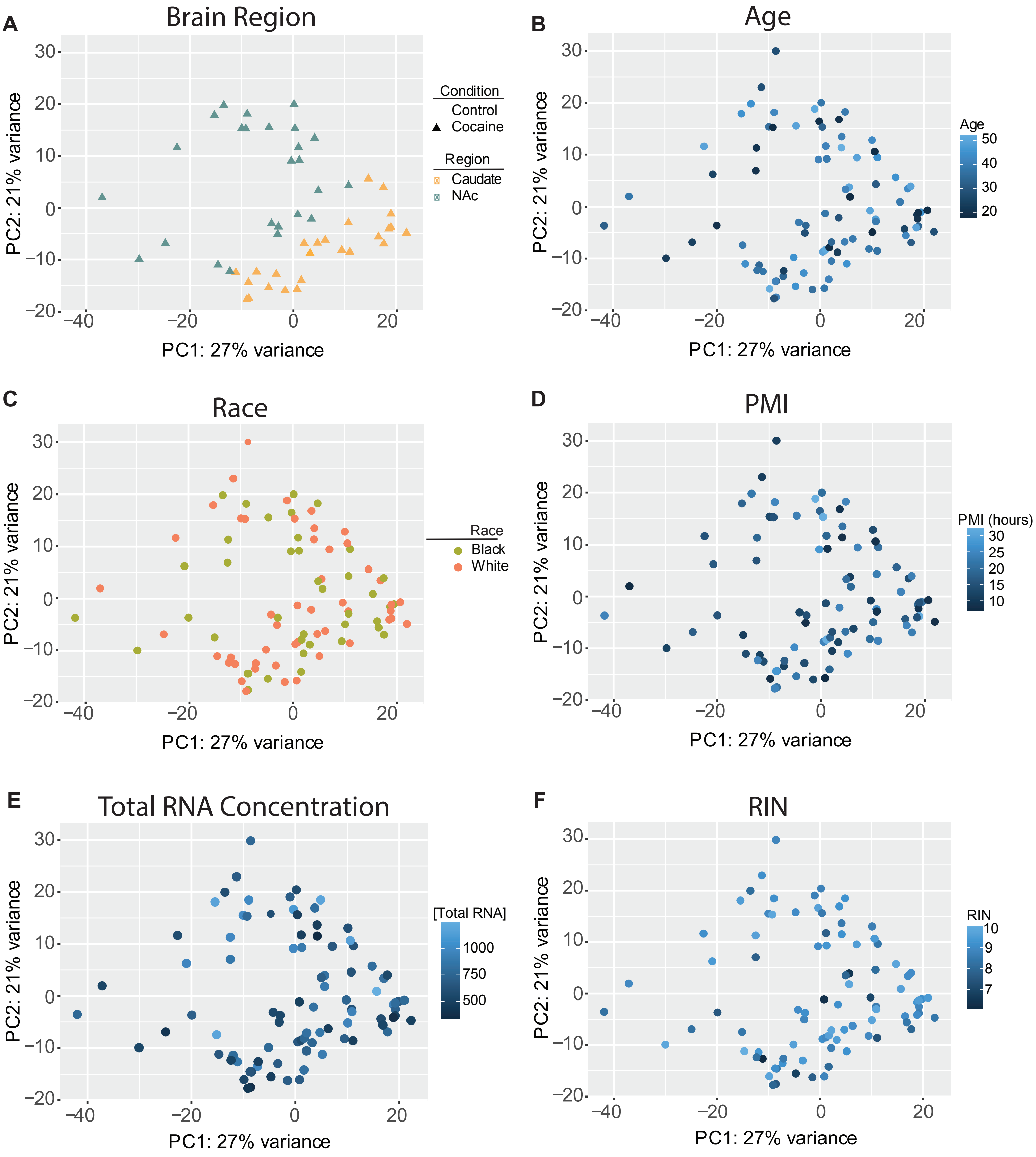

Figure S1. Examination of variables in human CUD and control cohort. Principal component (PC) analysis of the influence of data segregation of all samples based on several characteristics. Analysis demonstrated the influence of **(A)** brain region but not **(B)** age, **(C)** race, **(D)** postmortem internal (PMI), **(E)** total RNA concentration, or **(F)** RNA integrity number (RIN).

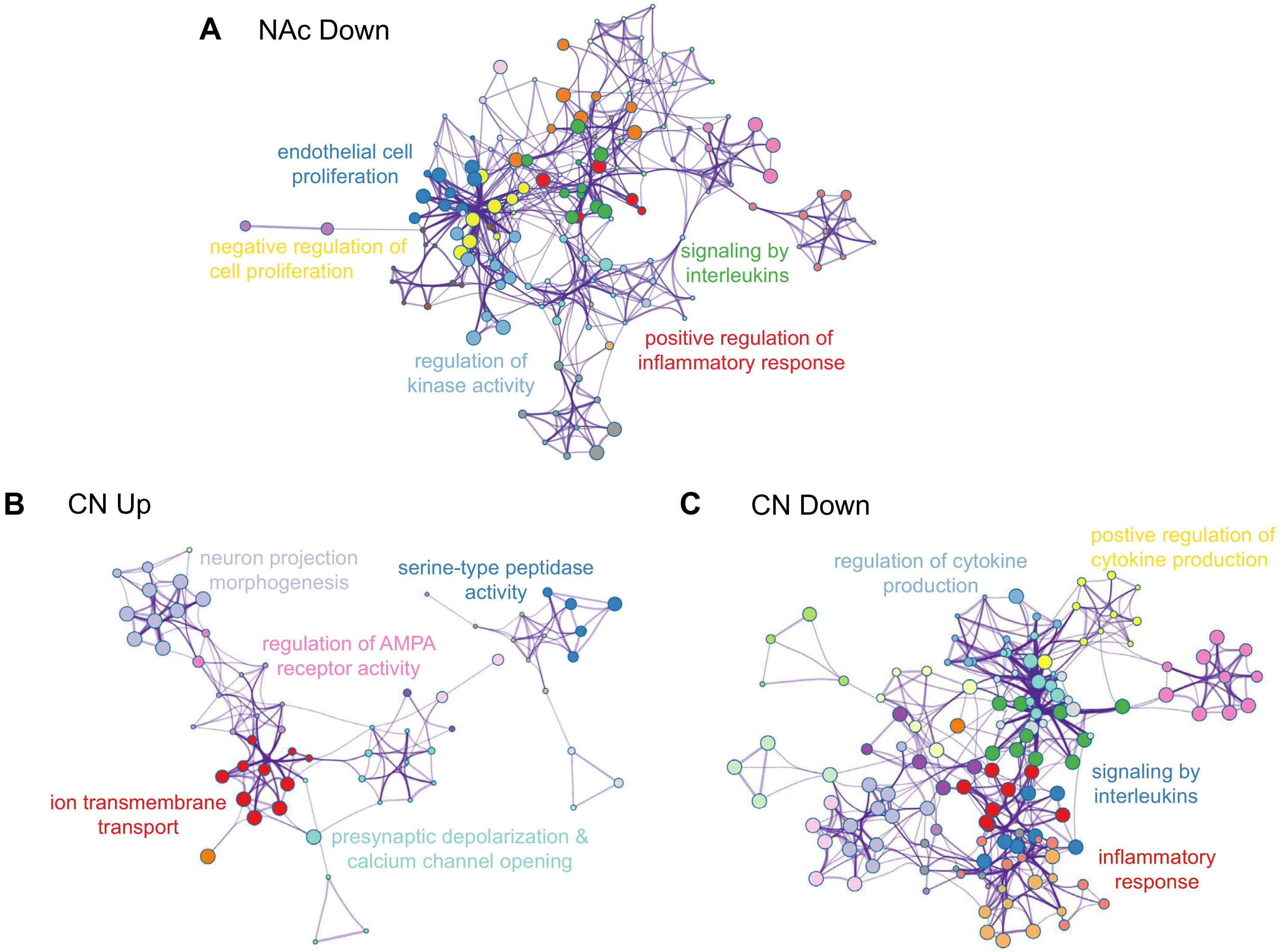

Figure S2. Visualization of GO term relationships driven by differentially expressed transcripts. (A) Network of GO terms enriched among transcripts that are downregulated in the NAc of people with CUD compared to control subjects, featuring processes related to synaptic plasticity and neural development. **(B)** Network of GO terms enriched among transcripts that are upregulated in the CN of people with CUD compared to control subjects, featuring a dense gene network of cell proliferation and neuroinflammatory processes. **(C)** Network of GO terms enriched among transcripts that are down in the CN of people with CUD compared to control subjects, featuring a dense gene network of neuroinflammatory processes.

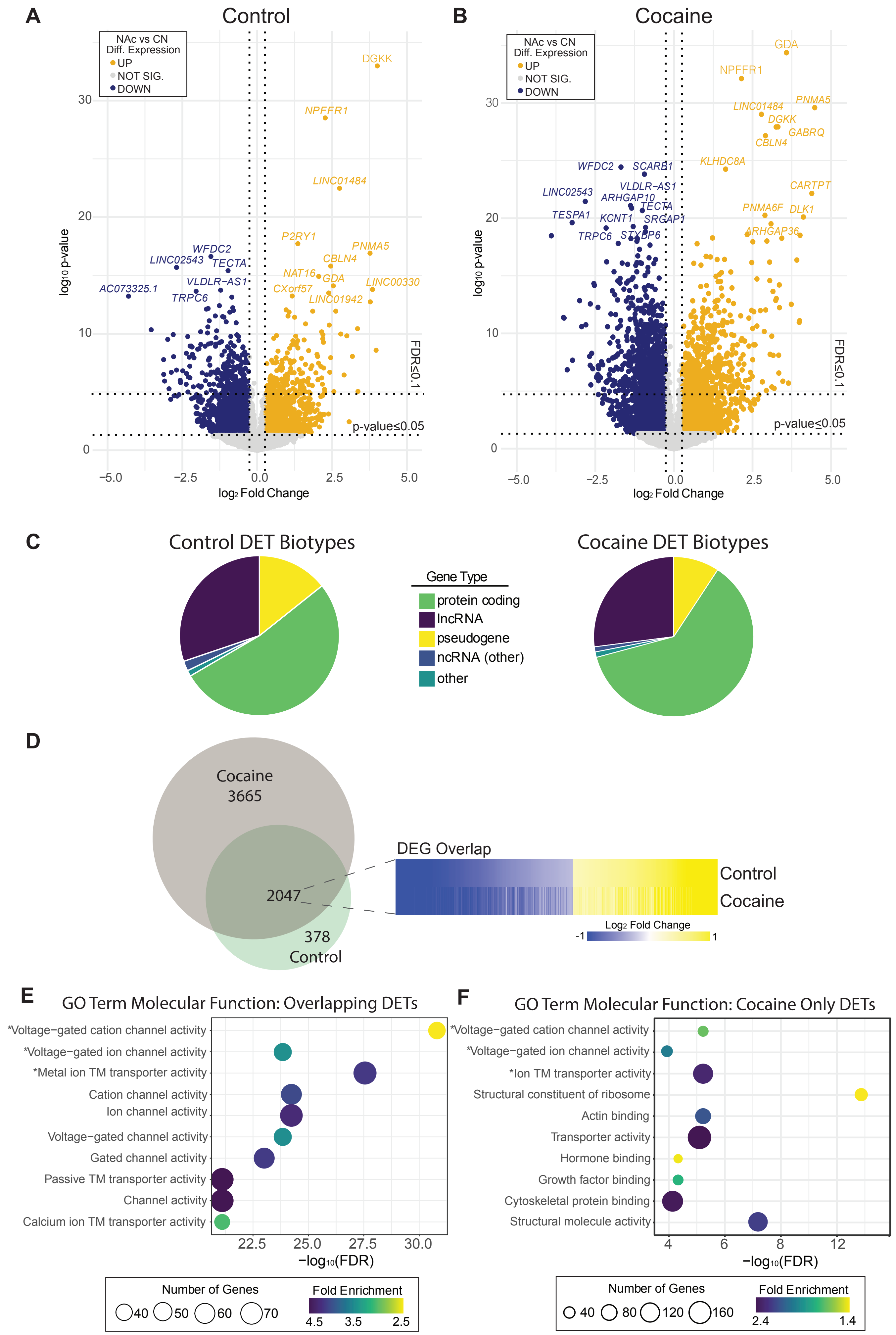
A
Control
NAc vs CN
Diff. Expression
UP
NOT SIG.
DOWN
DGKK
NPFFR1
LINC01484
P2RY1
PNMA5
CBLN4
NAT16
GDA
LINC00330
CXorf57
LINC01942
WFDC2
LINC02543
TECTA
VLDLR-AS1
AC073325.1
TRPC6
FDR≤0.1
p-value≤0.05
log10 p-value
log2 Fold Change
B
Cocaine
GDA
NPFFR1
PNMA5
LINC01484
DGKK
GABRQ
CBLN4
KLHDC8A
WFDC2
SCARB1
VLDLR-AS1
LINC02543
ARHGAP10
TECTA
CARTPT
PNMA6F
DLK1
TESPA1
KCNT1
SRGAP1
ARHGAP36
TRPC6
STXBP6
C
Control DET Biotypes
Cocaine DET Biotypes
Gene Type
protein coding
lncRNA
pseudogene
ncRNA (other)
other
D
Cocaine
3665
2047
378
Control
DEG Overlap
Control
Cocaine
Log2 Fold Change
E
GO Term Molecular Function: Overlapping DETs
*Voltage−gated cation channel activity
*Voltage−gated ion channel activity
*Metal ion TM transporter activity
Cation channel activity
Ion channel activity
Voltage−gated channel activity
Gated channel activity
Passive TM transporter activity
Channel activity
Calcium ion TM transporter activity
−log10(FDR)
Number of Genes
Fold Enrichment
F
GO Term Molecular Function: Cocaine Only DETs
*Voltage−gated cation channel activity
*Voltage−gated ion channel activity
*Ion TM transporter activity
Structural constituent of ribosome
Actin binding
Transporter activity
Hormone binding
Growth factor binding
Cytoskeletal protein binding
Structural molecule activity
−log10(FDR)
Number of Genes
Fold Enrichment

Figure S3. Transcriptomic differences between the NAc vs. CN from control subjects and from subjects with CUD. (A, B) Volcano plot showing DETs between NAc and CN in control subjects **(A)** and CUD subjects **(B)**. Log_2 plotted relative to log10 p-value with downregulated genes represented in blue and upregulated genes in yellow. Bottom horizontal dashed line represents a nominal p-value significance cutoff of $p \leq 0.05$ and the top horizontal dashed line represents an FDR significance cutoff of $q \leq 0.05$; vertical dashed lines represent Log_2FC cutoffs of ± 0.26 or (FC 1.2). **(C)** Biotypes of DETs between NAc and CN (nominal $p \leq 0.05$ and Log_2FC ± 0.26) in control subjects (left) and CUD subjects (right). Protein-coding genes represent the majority of DETs (green; 73.4%) followed by lncRNAs (purple; 19.5%). **(D)** Venn diagram comparing DETs between NAc and CN in control and CUD subjects shows no significant overlap between DETs in control and CUD individuals. Heatmap showing relative gene expression of overlapping genes indicates that DETs that overlap between CUD and healthy individuals show similar gene expression changes. Heatmap seeded by control samples (NAc vs CN).

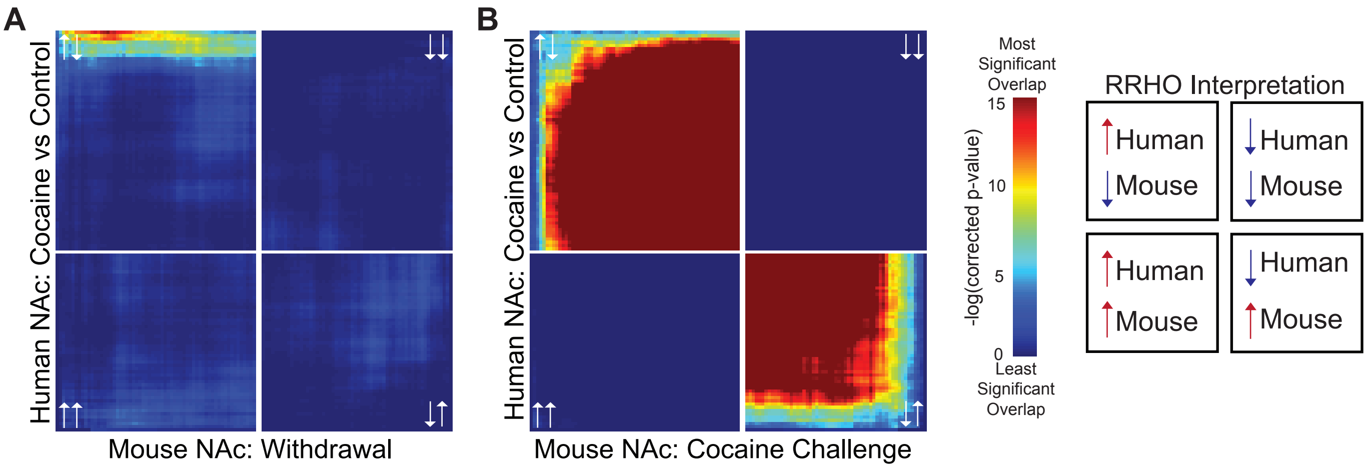

Figure S4. Transcriptional changes in human CUD brain regions compared to cocaine and saline challenge in mouse self-administration. Threshold-free RRHO analyses overlap in gene expression in the NAc of human CUD compared to mice that self-administered cocaine for 10 days, then withdrawn for 30 days, and exposed to a saline or cocaine challenge and euthanized 1 hr later *(17)*. **(A)** RRHO plot indicates lack of overlap in gene expression changes in NAc observed in withdrawal mice compared to CUD. **(B)** In contrast, RRHO plot indicates high overlap of opposite changes in NAc gene expression NAc of cocaine-challenged withdrawn mice compared to CUD. See Fig. 2A for definition of each quadrant.

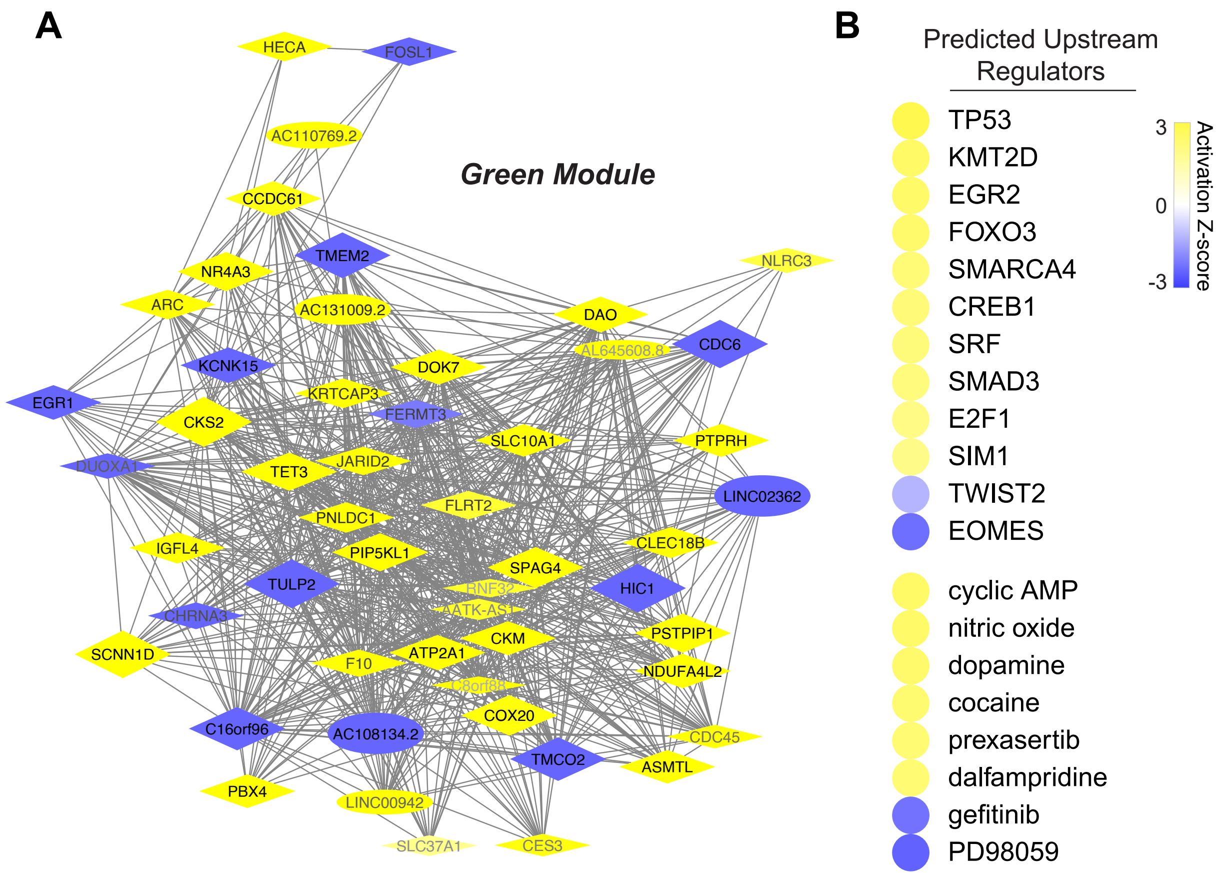

Figure S5. Co-expression gene networks non-conserved between human CUD and mouse cocaine self-administration. (A) Arachne plots show a human network that is not conserved in mouse self-administration, the human green module. **(B)** Predicted upstream regulators for the human green module.

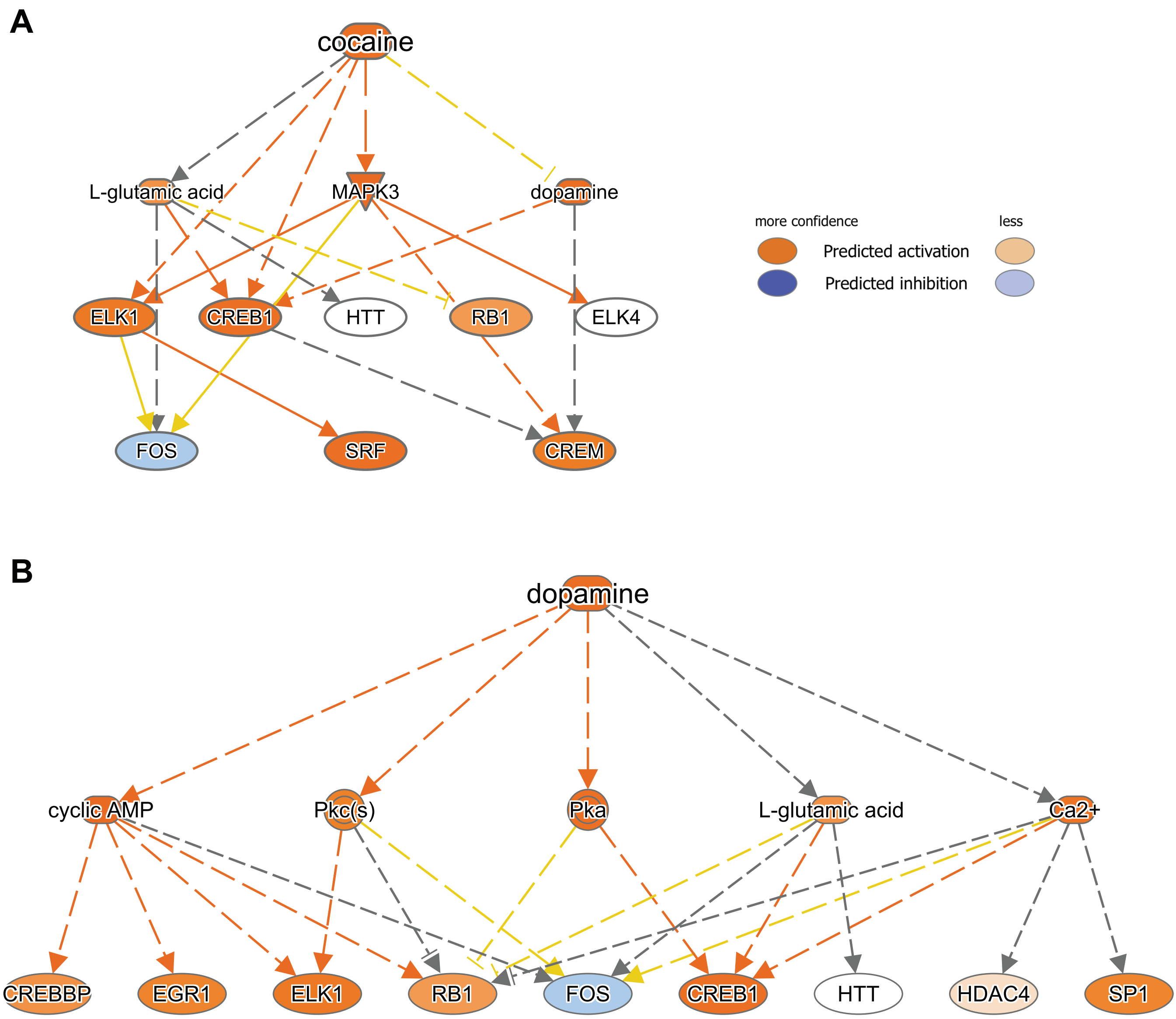

Figure S6. Cocaine and dopamine are upstream regulators of CUD-related gene expression in the human green network. (A) Mechanistic network of the predicted upstream regulator cocaine for CUD-related expression of the human green module (cocaine activation z-score = 2.25, p-val = 1.05e-05). **(B)** Mechanistic network of the upstream regulator dopamine for CUD-related expression of the human green module (dopamine activation z-score = 2.34, p-val = 1.22e-04). Mechanistic networks of the predicted regulators cocaine and dopamine generated by IPA *(67).*

Supplemental Tables

Table S1. Unaffected and CUD subjects' demographics and tissue characteristics. Values are n or mean ± SD; CUD, cocaine use disorder; PMI postmortem interval; RIN, RNA integrity number.

Table S2. Overlap of GWAS hits with NAc and CN differentially expressed genes. Differential expression statistics of genes in subjects with CUD ($p < 0.05$) that have been that have been previously implicated in CUD by GWAS (coloring according to the referenced GWAS study).

Table S3. WGCNA identified human gene co-expression networks in across CUD and control subjects. Module differential connectivity (MDC) scoring was used to detect CUD-specific modules that showed considerably more coordinated expression of transcripts, i.e., significantly higher connectivity, in subjects with CUD compared to control subjects. WGCNA identified 63 human co-expression modules, of which 16 networks showed greatly increased connectivity in CUD (MDC score > 2.5, FDR < 0.05).

Table S4. WGCNA identified mouse gene co-expression networks. WGCNA identified 61 mouse co-expression modules, of which 12 networks showed greatly increased connectivity in animals with a history of chronic cocaine intake compared to drug-naïve control mice (MDC score > 2.5, FDR < 0.05).

Table S5. Module conservation between humans and mice. Cross-species module conservation and their statistics by comparing module membership between the human and mouse cohorts.

Table S6. Cross-species module conservation of human CUD gene co-expression networks. Species module conservation between humans and mice shown for 16 human gene networks with increased connectivity in CUD (MDC score > 2.5, FDR < 0.05), which revealed 5 human CUD modules that were conserved in the mouse (Odds Ratio > 3.5, Bonferroni $p < 0.05$).
